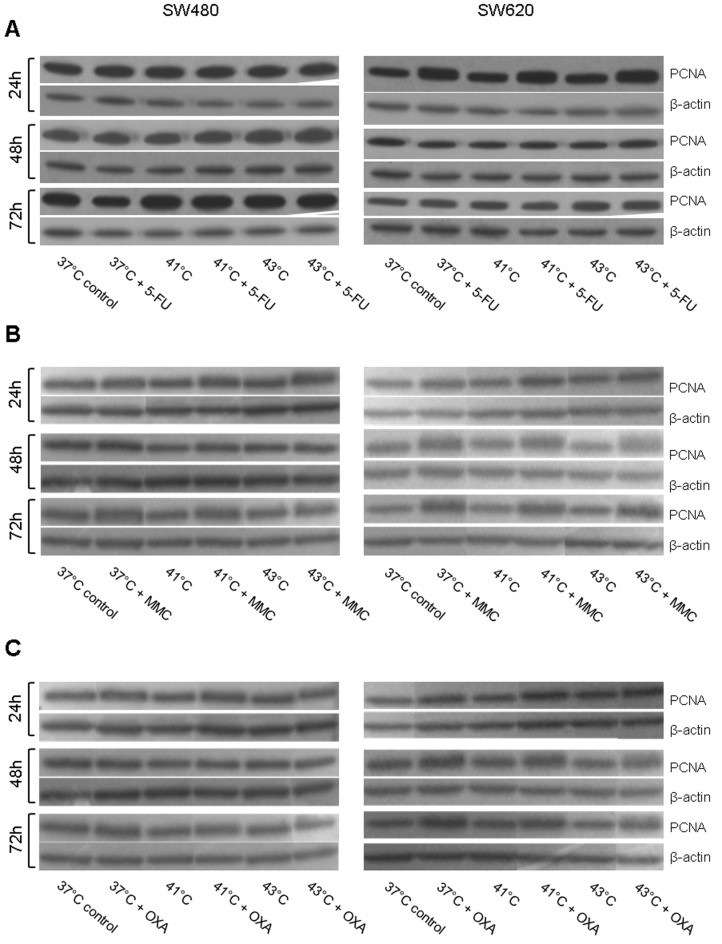# Upregulated Heat Shock Proteins After Hyperthermic Chemotherapy Point to Induced Cell Survival Mechanisms in Affected Tumor Cells From Peritoneal Carcinomatosis

**DOI:** 10.1177/1179064417730559

**Published:** 2017-09-18

**Authors:** Tanja Grimmig, Eva-Maria Moll, Kerstin Kloos, Rebecca Thumm, Romana Moench, Simone Callies, Jennifer Kreckel, Malte Vetterlein, Joerg Pelz, Buelent Polat, Sudipta Tripathi, Roberta Rehder, Carmen M Ribas, Anil Chandraker, Christoph-T Germer, Ana Maria Waaga-Gasser, Martin Gasser

**Affiliations:** 1 Department of Surgery I, Molecular Oncology and Immunology, University of Wuerzburg, Wuerzburg, Germany; 2 Division of Molecular Internal Medicine, Department of Internal Medicine II, University of Wuerzburg, Wuerzburg, Germany; 3 Department of Urology, University Medical Center Hamburg-Eppendorf, Hamburg, Germany; 4 Department of Surgery I, University of Wuerzburg, Wuerzburg, Germany; 5 Department of Radiation Oncology, University of Wuerzburg, Wuerzburg, Germany; 6 Transplant Research Center, Brigham and Women’s Hospital, Harvard Medical School, Boston, MA, USA; 7 Evangelical Medical School, Faculty University of Parana, Curitiba, Brazil.

**Keywords:** Heat shock proteins, peritoneal carcinomatosis, hyperthermic intraperitoneal chemotherapy, hyperthermia, apoptosis, chemoresistance

## Abstract

In patients with peritoneal carcinomatosis cytoreductive surgery combined with hyperthermic intraperitoneal chemotherapy (HIPEC) represents a promising treatment strategy. Here, we studied the role of hyperthermic chemotherapy on heat shock protein (HSP) expression and induction of tumor cell death and survival. HSP27, HSP70, and HSP90 combined with effects on tumor cell proliferation and chemosensitivity were analyzed in human colon cancer. Hyperthermic chemotherapy resulted in significant HSP27/HSP70 and HSP90 gene/protein overexpression in analyzed HT-29/SW480/SW620 colon cancer cells and peritoneal metastases from patients displaying amplified expression of proliferation markers, proliferating cell nuclear antigen and antiapoptotic protein Bcl-xL. Moreover, functionally increased chemoresistance against 5-fluorouracil/mitomycin C and oxaliplatin after hyperthermic chemotherapy points to induced survival mechanisms in cancer cells. In conclusion, the results indicate that intracellular HSP-associated antiapoptotic and proliferative effects after hyperthermic chemotherapy negatively influence beneficial effects of hyperthermic chemotherapy-induced cell death. Therefore, blocking HSPs could be a promising strategy to further improve the rate of tumor cell death and outcome of patients undergoing HIPEC therapy.

## Introduction

Peritoneal carcinomatosis (PC) is in strong context with morbidity and mortality in the management of several abdominal cancers, including colorectal, gastric, and ovarian cancers.^1^ Peritoneal seeding of cancer cells occurs in 10% to 20% of such patients with tumor being examined for potentially curative tumor resection.^2^ Because of the challenging treatment due to its relative resistance to systemic chemotherapy, PC has long been considered a terminal condition, and patients generally received palliative treatment.^3^ However, treatment strategies have improved over the past few decades and resulted in a combined strategy of cytoreductive surgery (CRS) of all macroscopic intraperitoneal tumors with hyperthermic intraperitoneal chemotherapy (HIPEC). Today, this combined treatment (CRS and HIPEC) is increasingly accepted as a therapeutic option in some primary and secondary peritoneal malignancies.^4^ Commonly used chemotherapeutic agents for HIPEC are mitomycin C (MMC) as well as oxaliplatin (OXA), the latter one with or without additional intravenous 5-fluorouracil (5-FU) treatment. Interestingly, in patients with PC from colorectal origin under such therapy, the 5-year overall survival rate was reported to range between 25% and 47%.^5–7^ As a consequence, an increasing number of tumor centers have been applying this combined therapeutic regimen.^8–13^

Heat shock proteins (HSPs) are highly conserved proteins found in all prokaryotic and eukaryotic cells. Expression levels of HSPs rise in response to different environmental, physical, and chemical stress factors including cytotoxic agents and hyperthermic conditions. Heat shock proteins play a crucial role in chaperoning misfolded proteins and protecting cells from damage and apoptosis.^14,15^ They interact with many proteins of cell signaling pathways that lead to reduced apoptosis and enhancement of cell migration, proliferation, and angiogenesis.^15–18^ In addition, overexpression of HSPs has been shown to be associated with poor prognosis in many tumor entities.^19,20^ Previously, we reported that HSP expression in tumor tissues of patients with PC from different tumor entities was highly upregulated after HIPEC and that hyperthermic conditions resulted in increased HSP expression and resistance to hyperthermia-induced cell death in human HT-29 colon cancer cells.^21^ Here, we demonstrate the effects of hyperthermic chemotherapy on HSP expression profiles, tumor cell proliferation, and chemosensitivity in several established human colon cancers using the well-established HIPEC protocols in humans.

## Materials and Methods

### Patients and human tissues

From a total of 63 patients who underwent CRS with additional HIPEC therapy as a closed procedure in our department (October 2008 to April 2011) and available representative tumor tissues before and after HIPEC therapy, 24 patients with isolated PC from colorectal cancer were investigated. Informed consent was obtained preoperatively, as was approved by the local medical ethics committee. Patient data are summarized in Table 1. The HIPEC therapy was performed under specific conditions (60 minutes permanent chemotherapeutical flux via external pump into the abdominal cavity after resection of relevant tumor masses with elevated temperature up to 41°C). Tumor biopsies from peritoneal tumors were taken before and after the HIPEC procedure. Tumor biopsies after a HIPEC procedure were taken from specifically marked peritoneal areas with small peritoneal metastases completely resected after the HIPEC. The samples were immediately frozen in liquid nitrogen until further protein extraction. Samples for subsequent RNA extraction were stored in RNAlater (Qiagen, Hilden, Germany). For cryostat sections, samples were embedded in Tissue-Tek O.C.T. Compound (Sakura, Torrance, CA, USA).

**Table 1.** Patients with PC from colorectal cancer assigned to cytoreductive surgery and hyperthermic intraperitoneal chemotherapy.

Gender	Age	UICC	pTNM	Grading	Primary/secondary PC	Follow-up, mo	Alive/death
Male	65	III	T4N0M0	3	S	26	D
	44	III	T4aN2bM0	3	S	36	A
	53	III	T4N1M0	2	S	39	A
	74	III	T4N1M0	2	S	26	D
	80	III	T4bN1bM0	2	S	11	A
	49	IV	T4aN1aM1	2	P	25	D
	44	IV	T3N2M1b	3	P	36	A
	49	IV	T4aN1bM1b	2	P	40	A
	56	IV	T4N1bM1b	2	P	18	D
	59	IV	T3N2bM1b	3	P	28	D
	67	IV	T4bN2M1b	2	P	32	D
Female	58	II	T3N0M0	3	S	48	A
	62	III	T4bN1aM0	2	S	46	A
	56	III	T2N2bM0	2	S	48	A
	38	III	T4aN1aM0	2	S	60	A
	37	III	T3N1bM0	2	S	36	A
	77	III	T4N1M0	2	S	15	A
	59	III	T4aN1M0	3	S	19	A
	59	IV	T3N0M1	2	P	20	D
	50	IV	T3N1cM1b	3	P	23	D
	56	IV	T4aN2aM1b	3	P	31	D
	41	IV	T3N2bM1	3	S	6	D
	41	IV	T3N2aM1b	2	P	25	A
	73	IV	T4aN2bM1b	3	P	30	A

Abbreviations: PC, peritoneal carcinomatosis; UICC, Union for International Cancer Control.

### Cell culture and in vitro HIPEC conditions

The human colon cancer cell lines HT-29, SW480, and SW620 were purchased from American Type Culture Collection (ATCC, Manassas, VA, USA) and cultured in RPMI 1640 medium (ATCC) supplemented with 10% fetal bovine serum and 1% penicillin/streptomycin at 37°C in 5% CO_2_. To mimic HIPEC-like conditions, cancer cells were incubated for 60 minutes at different temperatures (37°C, 41°C, and 43°C) including or without additional cytostatic treatment (5-FU: 11.5 µM, MMC: 25 µM, OXA: 150 µM) in a shaking water bath. Fresh normothermic medium without cytostatic agent was supplied afterward and cells were incubated for 30 minutes, 24, 48, and 72 hours before analysis.

### Immunofluorescence

Primary antibody against PCNA (proliferating cell nuclear antigen), HSP70, and Ki-67 were purchased from Abcam (Cambridge, UK); antibodies to HSP27 and HSP90 were obtained from Cell Signaling Technology (Danvers, MA, USA). Cy3 (indocarbocyanine) and fluorescein isothiocyanate conjugated secondary antibodies were obtained from Jackson ImmunoResearch (West Grove, PA, USA). The staining was performed on cryostat sections of snap frozen tumor specimen before and after HIPEC or on Cytospin specimen prepared 24 hours after exposure to hyperthermic chemotherapy. For immune staining, samples were fixed in acetone and incubated with the primary antibody in Tris-buffered saline plus 0.5% bovine serum albumin overnight at 4°C in a humidified chamber. Treatment with the secondary fluorochrome conjugated antibody was performed for 30 minutes at room temperature in a humidified chamber. Subsequently, slides were mounted with DAPI Fluoromount-G (Southern Biotech, Birmingham, AL, USA) and analyzed using an Olympus BX51 Microscope and the cellSens Dimension software (Hamburg, Germany).

### RNA extraction and quantitative real-time polymerase chain reaction

Gene expression of HSPB1 (HSP27-1), HSPA1A and HSPA1B (HSP70 1A and 1B), HSP90AA1 and HSP90AA2 (HSP90α A1 and A2) as well as proliferation markers PCNA and Ki-67 was determined using quantitative real-time polymerase chain reaction (PCR). Total cellular RNA was extracted using RNeasy Mini Kit (Qiagen) on the QIAcube platform (Qiagen) according to the manufacturer’s instructions. Reverse transcription was performed using ImProm II Reverse Transcriptase System (Promega, Mannheim, Germany) and Eppendorf Mastercycler (Eppendorf, Hamburg, Germany). Gene quantification was performed using RT^2^ qPCR Primer Assays (Qiagen) and MESA GREEN qPCR MasterMix (Eurogentec Deutschland GmbH, Koeln, Germany) according to the manufacturer’s protocol. Housekeeping genes β-actin and TBP (TATA-binding protein) were used for relative quantification. All PCR reactions were conducted in duplicates on a Biorad CFX96 Touch Real-Time PCR Detection System. Reproducibility was confirmed by 3 independent PCR runs. The relative gene expression value, fold difference (FD), is expressed as 2^−ΔΔCq^.

### Western blot

Protein extracts from tumor tissue samples and colon cancer cell lines were performed using lysis buffer radioimmunoprecipitation assay (RIPA)-containing 1,1,1-Trichloro-2,2-bis(4-chlorophenyl)ethane (DDT; Sigma-Aldrich, Steinheim, Germany) and protease/phosphatase inhibitor cocktails (Merck Millipore, Billerica, MA, USA). Tumor tissue samples before and after a HIPEC were cut in small pieces and homogenized for 10 minutes in lysis buffer using TissueLyser (Qiagen) before incubation on a rotator at 4°C for 30 minutes and subsequent centrifugation (full speed, 4°C, 20 minutes). The supernatant was collected and stored at −80°C. For preparation of cell lysates, adherent cells were detached using Accutase solution (Sigma-Aldrich) and pelleted at 300×*g* for 10 minutes. After resuspension in RIPA lysis buffer, cells were incubated on a rotator at 4°C for 30 minutes, then centrifuged (full speed, 4°C, 20 minutes), and supernatant was collected and stored at −80°C. Protein concentrations were determined by Bradford assay using Roti-Quant solution (Carl Roth, Karlsruhe, Germany). For sodium dodecyl sulfate polyacrylamide gel electrophoresis, NuPAGE SDS Buffer and NuPAGE Novex Mini Gels (Thermo Fisher Scientific) were used according to the manufacturer’s instructions. Western blotting on nitrocellulose was performed using iBlot Dry Blotting System and iBlot Gel Transfer Stacks (Thermo Fisher Scientific) according to the manufacturer’s protocol. Blots were probed with antibodies to HSP27, HSP70, HSP90, β-actin, Bcl-xL, and PCNA. Antibodies to HSP70 and Bcl-xL were obtained from Abcam. HSP27, HSP90, PCNA, and β-actin antibodies were purchased at Cell Signaling Technology. Anti-mouse IgG and anti-rabbit IgG horseradish peroxidase (HRP)-conjugated secondary antibodies were obtained from Santa Cruz Biotechnology (Dallas, TX, USA). Quantification was performed using ImageJ software: relative optical density (ROD) was expressed as values for proteins of interest in relation to values of β-actin loading controls.

### MTS proliferation and chemosensitivity assay

To investigate the effect of hyperthermia on tumor cell proliferation and chemoresistance, 4000 cells/well (HT-29) or 8000 cells/well (SW480, SW620) were seeded in 96 well plates and preincubated for 3 days. To mimic HIPEC-like conditions, cells were treated as described previously. Cell viability was measured using CellTiter 96 AQueous One Solution Cell Proliferation Assay (Promega, Mannheim, Germany) according to the manufacturer’s instructions.

### HSP inhibition assay

As described previously, 4000 cells/well (HT-29) or 8000 cells/well (SW480, SW620) were seeded in 96-well plates and preincubated for 3 days. The HIPEC-like conditions were then applied as defined above. Immediately after exposure to hyperthermic chemotherapy, cells were treated with a combination of HSP90 and HSP70 inhibitors, as single HSP90 inhibition is known to induce the activation of the heat shock response, a cell survival mechanism that induces overexpression of HSP7070 and other HSPs.^22^ About 500 mM 17-AAG (HSP90 inhibitor; Santa Cruz Biotechnology) and 10 µM VER155008 (HSP70 inhibitor) were applied and cell viability was measured 24 hours after exposure as described before.

## Results

### Western blot analysis of HSP27, HSP70, and HSP90 after hyperthermic chemotherapy

**Figure 1.**
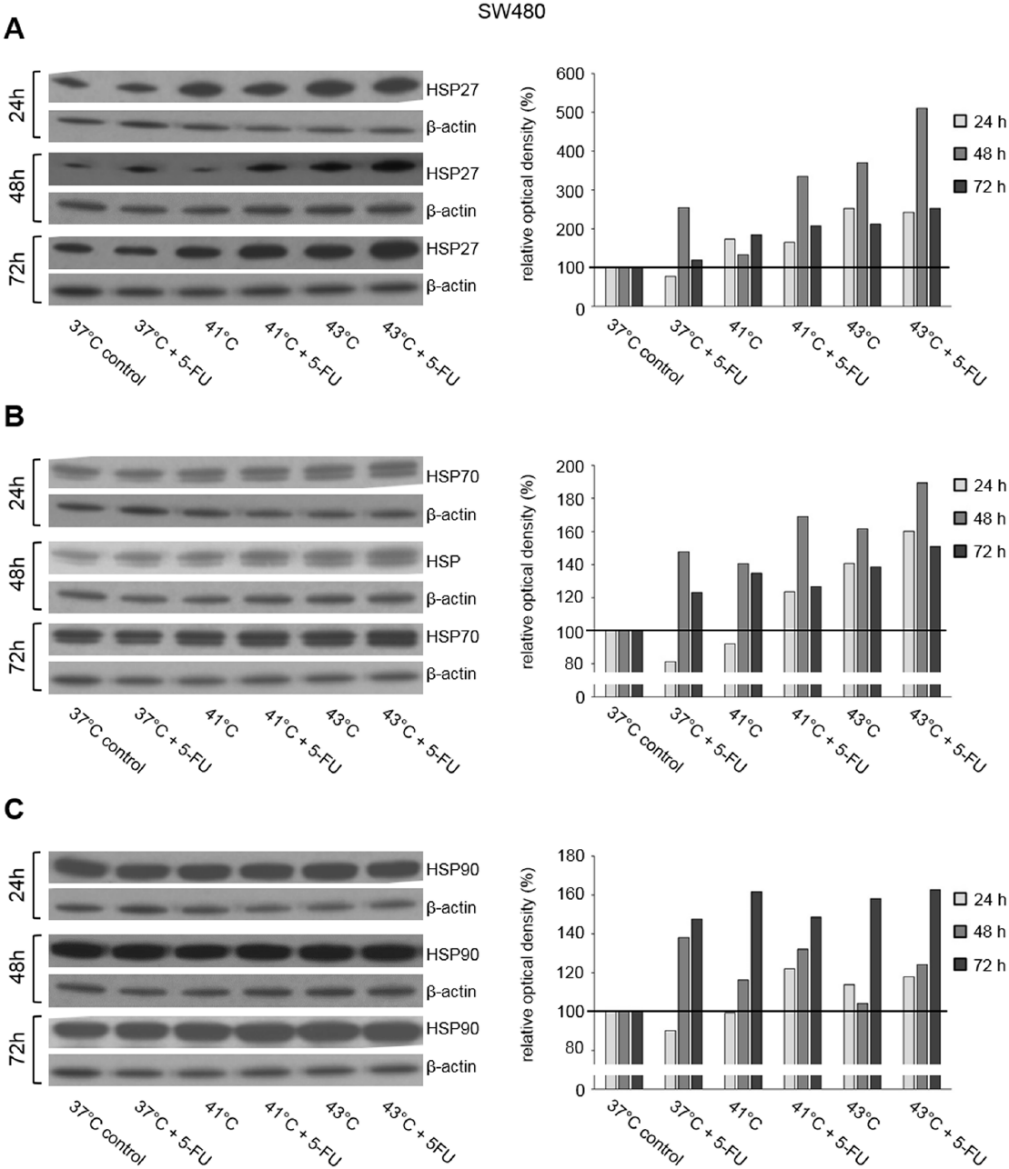
Western blot analysis of (A) HSP27, (B) HSP70, and (C) HSP90 in SW480 cells 24, 48, and 72 hours after treatment (60 minutes) with hyperthermia including or without additional chemotherapy using 5-fluorouracil (5-FU). β-actin probe was used as a control for protein loading. Relative optical density was determined using ImageJ software: values for proteins of interest were calculated in relation to values of loading controls. Normothermic cells (37°C control) were standardized to baseline.

**Figure 2.**
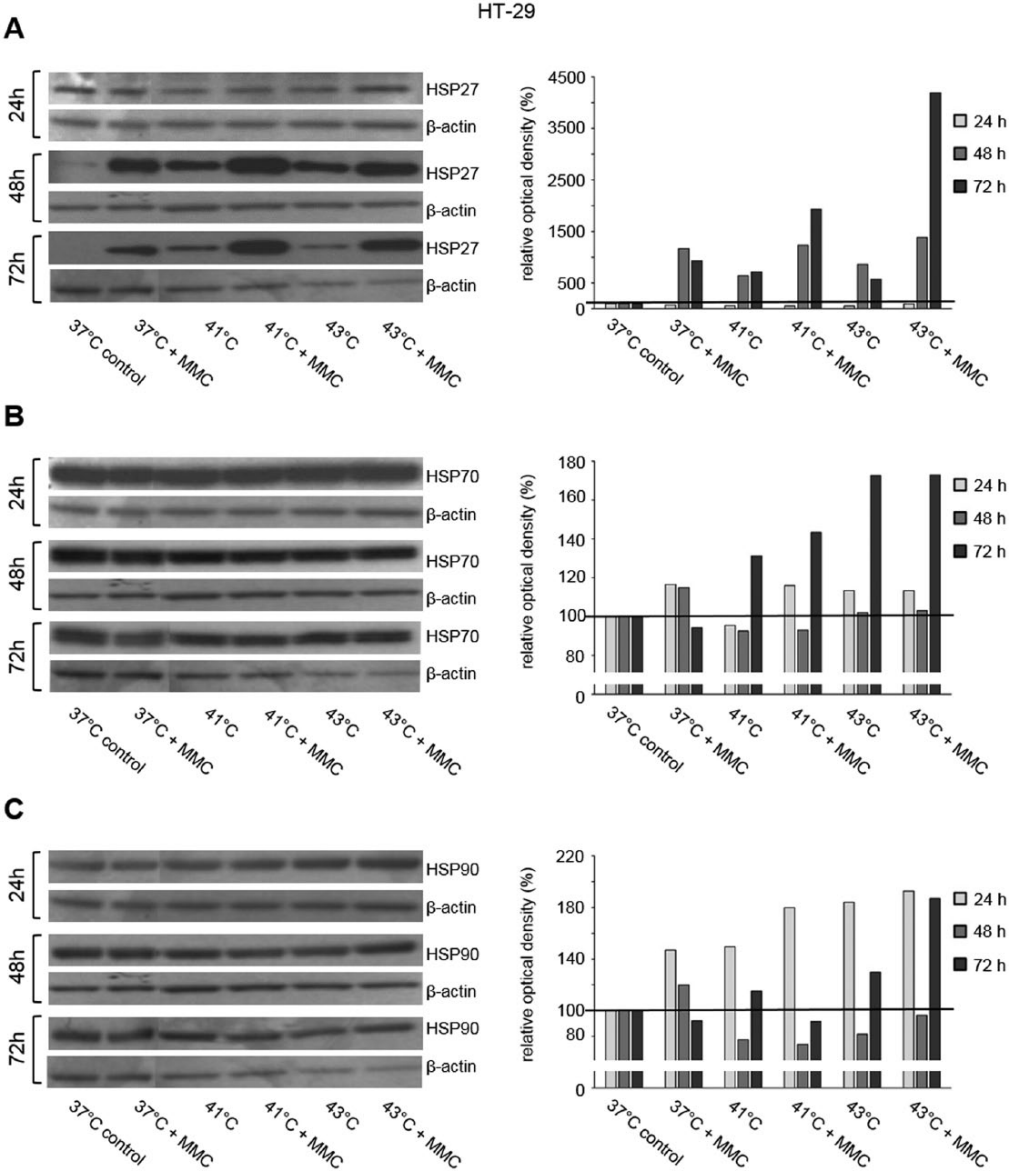
Western blot analysis of (A) HSP27, (B) HSP70, and (C) HSP90 in HT-29 cells 24, 48, and 72 hours after treatment (60 minutes) with hyperthermia including or without additional chemotherapy using mitomycin C (MMC). β-actin probe was used as a control for protein loading. Relative optical density was determined using ImageJ software: values for proteins of interest were calculated in relation to values of loading controls. Normothermic cells (37°C control) were standardized to baseline.

**Figure 3.**
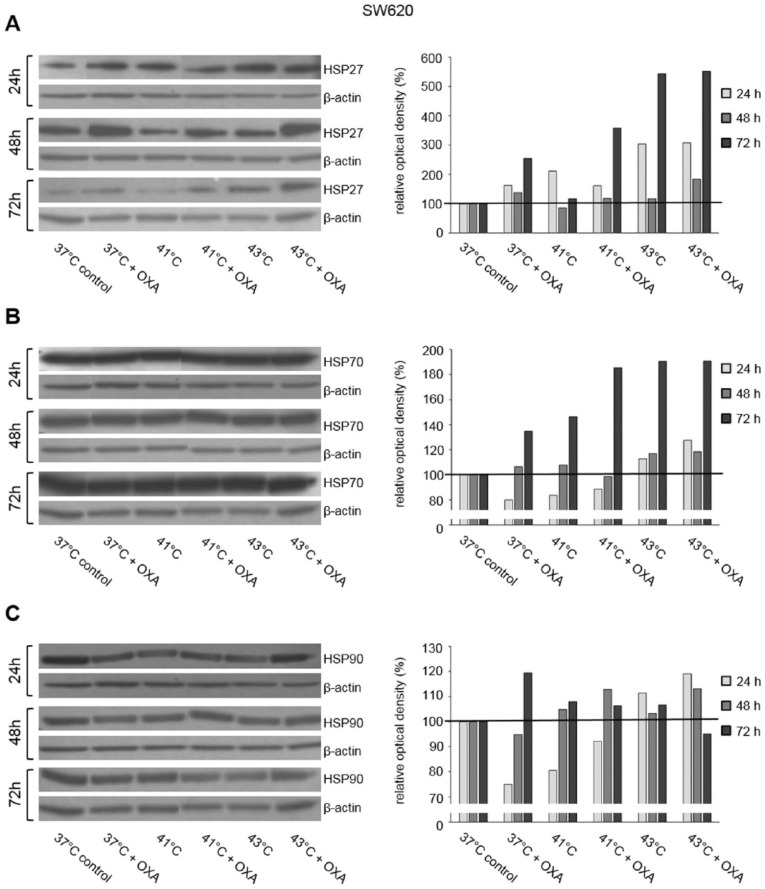
Western blot analysis of (A) HSP27, (B) HSP70, and (C) HSP90 in SW620 cells 24, 48, and 72 hours after treatment (60 minutes) with hyperthermia including or without additional chemotherapy using oxaliplatin (OXA). β-actin probe was used as a control for protein loading. Relative optical density was determined using ImageJ software: values for proteins of interest were calculated in relation to values of loading controls. Normothermic cells (37°C control) were standardized to baseline.

HSP27, HSP70, and HSP90 Western blot analysis was performed in 3 human colon cancer cell lines (HT-29, SW480, and SW620) 24, 48, and 72 hours after exposure to hyperthermia with or without additional chemotherapy using 5-FU, MMC, and OXA. Representative Western blots of this detailed protein analysis are demonstrated in Figures 1 to 3.

In SW480 colon cancer cells, increased HSP27 protein expression was observed 1, 2, and 3 days after initial hyperthermia (60 minutes) compared with normothermic controls (41°C and 43°C versus 37°C) (Figure 1A). Additional exposure to 5-FU caused further increase in HSP27 protein expression at all investigated time points (Figure 1A). Similar HSP27 expression profiles were obtained in HT-29 and SW620 colon cancer cells (Supplementary Figure S1A). Although 24 hours after incubation with or without MMC no relevant changes in HSP27 expression were detected in HT-29 cells, upregulated expression as in SW480 for 5-FU tumor cells was demonstrated after 48 and 72 hours: hyperthermia alone resulted in intense HSP27 expression compared with normothermic controls and additional exposure to MMC caused further increase in expression (Figure 2A). SW620 cancer cells showed upregulated HSP27 expression 24 hours after treatment with hyperthermia alone, whereas additional chemotherapy with OXA led to increased protein expression even after 72 hours (Figure 3A). Similar effects of chemotherapy were also detected in HT-29 (OXA) and SW620 and SW480 colon cancer cells, respectively (MMC: Supplementary Figure S1B and OXA: Supplementary Figure S1C).

Although HSP27 expression was detected at moderate basal levels and was strongly inducible through hyperthermia and/or cytostatic treatment, intense HSP70 and HSP90 baseline expression was already observed in all investigated normothermic controls (HT-29, SW480, and SW620 colon cancer cells). Further increase in HSP70 expression after hyperthermic chemotherapy was demonstrated in tumor cells exposed to 5-FU, MMC, and OXA (Figures 1B, 2B, and 3B), whereas HSP90 expression was found elevated only after treatment with 5-FU and MMC (Figures 1C and 2C).

To additionally visualize HSP expression increase after exposure to chemotherapy, representative examples of immunofluorescent stainings in SW480 cells treated with 5-FU at normothermia (37°C) and hyperthermia (41°C and 43°C) are demonstrated in Figure 8 (red fluorescence).

### HSP gene expression profiles after hyperthermic chemotherapy

Based on the results of the Western blot analysis, the induction of HSP27, HSP70, and HSP90 messenger RNA (mRNA) expression was further analyzed using 2 different isoforms for both HSP70 and HSP90 (HSPA1A: HSP70 1A and HSPA1B: HSP70 1B; HSP90AAA1: HSP90α A1 and HSP90AA2: HSP90α A2).

**Figure 4.**
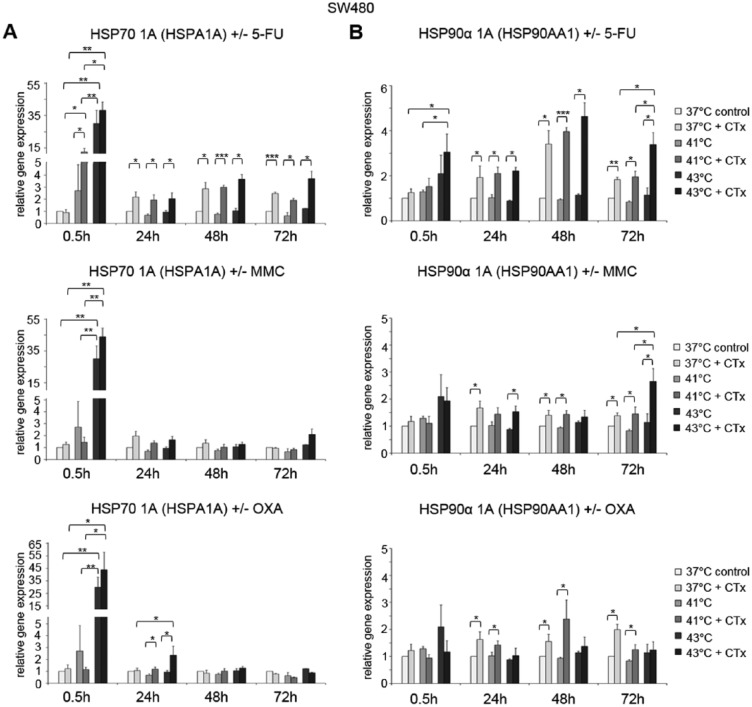
(A) Gene expression analysis (real-time polymerase chain reaction) of HSPA1A (HSP70 1A) and (B) HSP90AA1 (HSP90α 1A), in SW480 cells 0.5, 24, 48, and 72 hours after treatment (60 minutes) with hyperthermia including or without additional chemotherapy (CTx) using 5-fluorouracil (5-FU), mitomycin C (MMC), or oxaliplatin (OXA). Normothermic cells without cytostatic treatment (37°C control) were standardized to baseline. The relative gene expression is expressed as 2^−ΔΔCq^ (= fold difference, FD). Reproducibility was confirmed by 3 independent runs. *P < .05; **P < .0005; ***P < .0001 (Student *t* test).

HSP70 expression was significantly increased in SW480 colon cancer cells early after exposure to hyperthermia and particularly after profound hyperthermia of 43°C (HSP70 1A [HSPA1A] at 0.5 hours: 41°C = FD 2.7, 43°C = FD 30.0 versus normothermic control = FD 1) (Figure 4A, top). Additional chemotherapy with 5-FU resulted in further increased gene expression (41°C = FD 12.6 and 43°C = FD 38). After 24, 48, and 72 hours, significantly upregulated HSP70 1A [HSPA1A] mRNA expression levels were observed in 5-FU–treated cancer cells at all investigated temperatures (37°C: FD 2.2, FD 2.9, and FD 2.5; 41°C: FD 1.9, FD 3.0, and FD 1.9; 43°C: FD 2.1, FD 3.7, and FD 3.7, respectively) (Figure 4A, top). Comparable results of upregulated HSP70 expression were found after exposure to MMC at the initial time point (0.5 hours), although expression returned to control levels later on (Figure 4A, center). When exposed to OXA, significantly increased HSP70 1A [HSPA1A] gene expression was demonstrated again at the initial time point (0.5 hours: FD 43.9) as well as the next interval (24 hours: FD 2.4) in SW480 cancer cells. However, no gene expression changes were observed at later time points (Figure 4A, bottom). HT-29 and SW620 colon cancer cells treated with chemotherapy demonstrated comparable effects on HSP70 1A [HSPA1A] (5-FU and MMC: Supplementary Figure S2A and 5-FU, MMC, and OXA: Supplementary Figure S2B). In accordance with these results, comparable findings were obtained for HSP70 1B [HSPA1B] (representative example in SW480 cells: Supplementary Figure S3A).

HSP90α A1 [HSP90AA1] gene expression was found to be significantly increased in SW480 cells at investigated initial high temperatures (41°C and 43°C) and time points when treated with 5-FU and compared with normothermic controls and cells without chemotherapy (Figure 4B, top). Early after hyperthermic incubation at 43°C with 5-FU (0.5 hours), tumor cells showed a 3-fold increased gene expression. After 24, 48, and 72 hours, 5-FU–treated colon cancer cells again demonstrated significantly upregulated mRNA expression when incubated under normothermic and hyperthermic conditions (37°C: FD 1.9, FD 3.4, and FD 1.8, respectively; 41°C: FD 2.1, FD 4.0, and FD 2.0, respectively; and 43°C: FD 2.2, FD 4.6, and FD 3.4, respectively) (Figure 4B, top). Interestingly, when compared with results after 24 hours, cells exposed to 5-FU at 43°C showed highest mRNA expression at all time points. In cancer cells treated with MMC, again significantly increased HSP90α A1 [HSP90AA1] expression profiles were detected at 43°C (24 hours: FD 1.7, 48 hours: FD 1.4, and 72 hours: FD 1.4) as well as both hyperthermic temperatures (41°C = 48 hours: FD 1.4, 72 hours: FD 1.5; 43°C = 24 hours: FD 1.5, 72 hours: FD 2.6) (Figure 4B, center), whereas SW480 cells treated with OXA showed upregulated expression after normothermic incubation (24 hours: FD 1.6, 48 hours: FD 1.2, 72 hours: FD 2.0) and hyperthermic condition at 41°C (24 hours: FD 1.4, 48 hours: FD 2.4, 72 hours: FD 1.3) (Figure 4B, bottom). Comparable results were obtained in colon cancer cell lines HT-29 and SW620 (Supplementary Figure S4A and S4B). In addition, representative comparable results of expression analysis of HSP90α A2 [HSP90AA2] in SW620 colon cancer cells are demonstrated in Supplementary Figure S3B.

**Figure 5.**
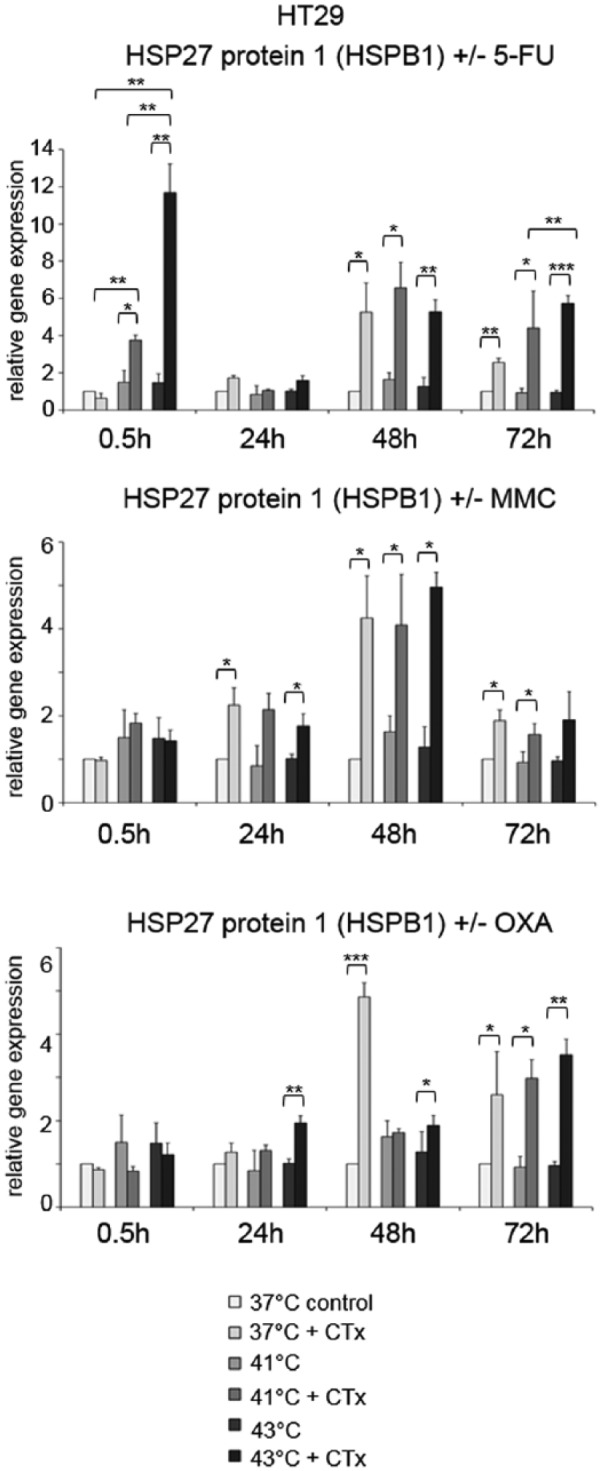
Gene expression analysis (real-time polymerase chain reaction) of HSPB1 (HSP27 1) in HT-29 cells 0.5, 24, 48, and 72 hours after treatment (60 minutes) with hyperthermia including or without additional chemotherapy (CTx) using 5-fluorouracil (5-FU), mitomycin C (MMC), or oxaliplatin (OXA). Normothermic cells without cytostatic treatment (37°C control) were standardized to baseline. The relative gene expression is expressed as 2^−ΔΔCq^ (= fold difference, FD). Reproducibility was confirmed by 3 independent runs. *P < .05; **P < .0005; ***P < .0001 (Student *t* test).

Next to HSP70 and HSP90 gene expression, the profiles of HSP27 (HSP27-1, HSPB1) were analyzed. HSP27 was significantly overexpressed in HT-29 colon cancer cells early after exposure to hyperthermic chemotherapy with 5-FU compared with normothermic controls and tumor cells without chemotherapy (0.5 hours after 41°C: FD 3.8 and 43°C: FD 11.7 versus controls without cytostatic treatment 41°C: FD 1.5 and 43°C: FD 1.5) (Figure 5, top). No changes in HSP27 gene expression were detected 24 hours after hyperthermic chemotherapy, whereas 48 and 72 hours after incubation, significantly upregulated expression was again observed in HT-29 cancer cells treated with 5-FU at normothermic and hyperthermic conditions (37°C: FD 5.3 and FD 2.6, 41°C: FD 6.6 and FD 4.4, and 43°C: FD 5.3 and FD 5.7, respectively) (Figure 5, top). When incubated with MMC, significantly elevated HSP27 expression was again observed under normothermic and hyperthermic conditions 24 and 48 hours after exposure (37°C: FD 2.3 and FD 4.3, 41°C: FD 2.1 and FD 4.1, and 43°C: FD 1.8 and FD 5.0, respectively) (Figure 5, center). When treated with OXA, again significantly upregulated expression was demonstrated at 24, 48, and 72 hours (37°C: FD 4.4 and FD 2.5, 41°C: FD 3.0, and 43°C: FD 2.1, FD 2.0, and FD 3.6, respectively) (Figure 5, bottom). Next to HT-29, SW480, and SW620 colon cancer cells treated with chemotherapy demonstrated comparable effects (5-FU: Supplementary Figure S5A and 5-FU and MMC: Supplementary Figure S5B).

A comprehensive overview of the HSP gene expression changes in HT-29 colon cancer cells is given in Table 2 to allow for comparison of heat stress–induced and chemotherapy-induced HSP upregulation and for differentiation on the background of the 3 commonly used cytotoxic agents and temperatures. This demonstrates highly upregulated HSPs very early after hyperthermic but not normothermic chemotherapy (>2-fold, Table 2) (0.5 hours after treatment, Table 2). After 48 and 72 hours of initial treatment with cytostatic and chemotoxic substances, tumor cells showed upregulated HSP expression profiles (>2-fold), which were mostly independent of initially used normothermic or hyperthermic conditions. This suggests rather chemotoxicity-induced intracellular stress mechanisms over time than hyperthermia-mediated molecular changes. Comparing the used cytostatic/cytotoxic agents, treatment with 5-FU interestingly resulted in most distinct mRNA upregulation in all investigated HSPs and at most time points, whereas MMC showed moderate influence on HSP induction over time (Table 2). Differences between the used cytostatic agents may occur due to their different modes of action. While 5-FU is incorporated in DNA and RNA thus inhibiting transcription and translation, MMC serves as a prototype for drugs with bioreductive alkylation and OXA binds to DNA bases, thus leading to cross-linking of and inhibiting DNA synthesis and transcription.^23–25^

**Table 2.** HSP gene expression in HT-29 colon cancer cells after treatment with normothermic and hyperthermic chemotherapy.

Conditions	Time, h	HSP70 1A (HSPA1A)			HSP90a IA (HSP9OAA1)			HSP27-1 (HSPB1)		
		5-FU	MMC	OXA	5-FU	MMC	OXA	5-FU	MMC	OXA
37°C + CTx	0.5	−	−	+	−	−	+	−	−	−
41°C + CTx	0.5	++++	+++	+	++	++	−	++	+	−
43°C + CTx	0.5	+++++	+++	+++	++	++	−	+++	+	+
37°C + CTx	24	+	+	+	+	+	+	+	++	+
41°C + CTx	24	−	+	−	+	+	+	+	++	+
43°C + CTx	24	+	+	+	++	+	+	+	+	+
37°C + CTx	48	+	−	++	++	+	++	+++	++	++
41°C + CTx	48	+	+	−	++	++	+	+++	++	+
43°C + CTx	48	++	−	+	++	+	+	+++	++	+
37°C + CTx	72	++	−	+	++	+	+	++	+	++
41°C + CTx	72	++	−	++	++	−	+	++	+	++
43°C + CTx	72	++	−	+	+++	−	+	+++	+	++

Abbreviations: 5-FU, 5-fluorouracil; CTx chemotherapy; HSP, heat shock protein; MMC, mitomycin C; OXA, oxaliplatin.

HSP gene expression (quantitative real-time PCR) after treatment (60 minutes) with hyperthermia including or without additional CTx using 5-FU, MMC, or OXA. Normothermic cells (37°C) were standardized to baseline (= 1). Gene expression is as follows: − <1-fold, + >1-fold, ++ >2-fold, +++ >5-fold, ++++ >15-fold, and +++++ >30-fold difference.

### Effects of hyperthermic chemotherapy on tumor cell proliferation

**Figure 6.**
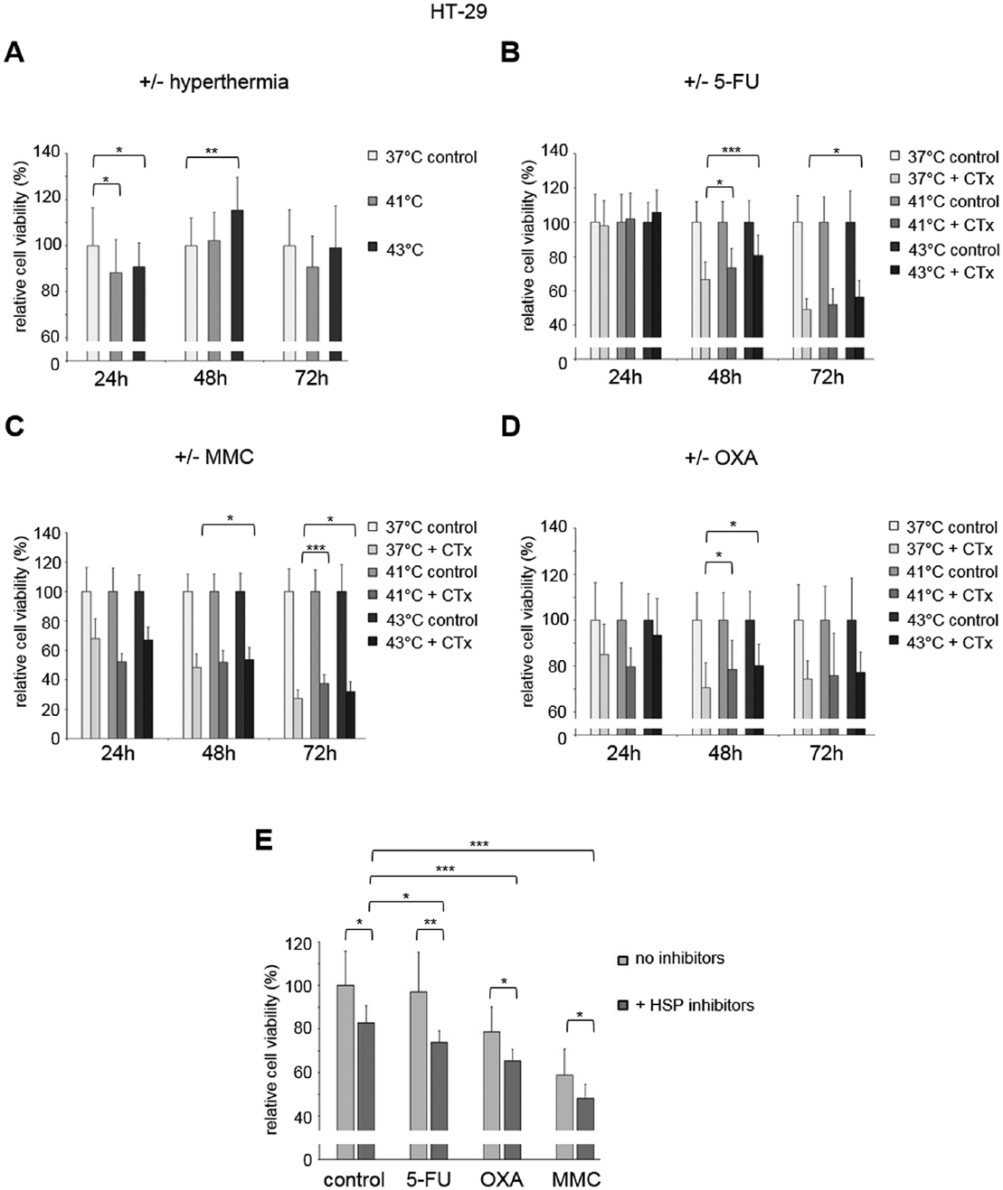
(A) MTS proliferation assay of HT-29 colon cancer cells 24, 48, and 72 hours after single hyperthermic treatment (60 minutes) or (B) additional chemotherapy (CTx) during hyperthermic intraperitoneal chemotherapy–like conditions using 5-fluorouracil (5-FU), (C) mitomycin C (MMC), or (D) oxaliplatin (OXA). (E) MTS proliferation assay of HT-29 colon cancer cells 24 hours after hyperthermic chemotherapy (43°C) including or without additional combined HSP70/90 inhibition using 17-AAG (HSP90) and VER155008 (HSP70). Colon cancer cells under normothermic condition (A, 37°C control), colon cancer cells without cytostatic treatment (B-D, control), and colon cancer cells without exposure to cytostatic agents and HSP inhibitors (E, control), respectively, were standardized to baseline. *P < .05; **P < .0005; ***P < .0001 (Student *t* test).

To dissect the effects of hyperthermia and hyperthermic chemotherapy on tumor cell growth, MTS proliferation assays were performed. HT-29 cancer cells showed decreased cell viability 24 hours after isolated hyperthermic treatment compared with tumor cells incubated under normothermic condition (41°C: 88% and 43°C: 91% versus 37°C: 100%, each *P* < .05), whereas 48 hours after hyperthermic incubation, significantly elevated cell viability was observed (43°C: 115%, *P* < .005) suggesting a rebound effect in cell proliferation (Figure 6A). After 3 days of exposure to hyperthermia, cell viability returned to normal (Figure 6A).

Interestingly, HT-29 tumor cells that were incubated with 5-FU under hyperthermic condition showed increased cell viability compared with normothermic incubation with 5-FU (48 hours after treatment at 41°C: 78%, and 43°C: 81%, versus 37°C: 66%, *P* < .05 and *P* < .005, respectively, and after 72 hours at 43°C: 56% versus 37°C: 49%, *P* < .05) (Figure 6B). Hyperthermic incubation with MMC demonstrated comparable results: cell viability of surviving HT-29 cells at lower level when compared with 5-FU treatment was significantly increased after 48 hours (43°C: 54% versus 37°C: 48%, *P* < .05) and after 72 hours (41°C: 37% and 43°C: 32% versus 37°C: 27%, *P* < .0001 and *P* < .05, respectively) (Figure 6C). Moreover, treatment with OXA under hyperthermic condition again resulted in increased cell viability 48 hours after initial incubation with the drug (41°C: 79% and 43°C: 81% versus 37°C: 71%, each *P* < .05) (Figure 6D). Hyperthermic treatment of SW480 colon cancer cells with 5-FU, MMC, and OXA demonstrated comparable results 24 hours after drug exposure and thus confirmed the observation of increased cell viability of surviving cancer cells (Supplementary Figure S6).

### Effects of HSP inhibition after hyperthermic chemotherapy

To analyze the potential benefit of additional HSP inhibition, a combination of HSP90 (17-AAG) and HSP70 (VER155008) was applied to HT-29 cancer cells after exposure to hyperthermic chemotherapy at 43°C. Colon cancer cells exposed to simultaneous HSP90/HSP70 inhibition demonstrated significantly decreased cell viability compared with samples without inhibitors in control cells and tumor cells pretreated with 5-FU, OXA, or MMC (control cells: 83% versus 100%, *P* < .05; 5-FU: 97% versus 77%, *P* < .005; OXA: 79% versus 66%, *P* < .05; MMC: 59% versus 48%, *P* < .05) (Figure 6E). Interestingly, when comparing HSP inhibitor–treated control cells with HT-29 cells additionally pretreated with 5-FU, OXA, or MMC, cell viability was significantly reduced in cells previously exposed to cytostatic agents (5-FU: 83% versus 77%, *P* < .05; OXA: 83% versus 66%, *P* < .0001; MMC: 83% versus 48%, *P* < .0001) (Figure 6E).

### Cellular expression of proliferation markers after hyperthermic chemotherapy

Next to cell viability, chemotoxic and heat stress–mediated effects on expression of proliferation markers PCNA and Ki-67 were investigated.

**Figure 7.**
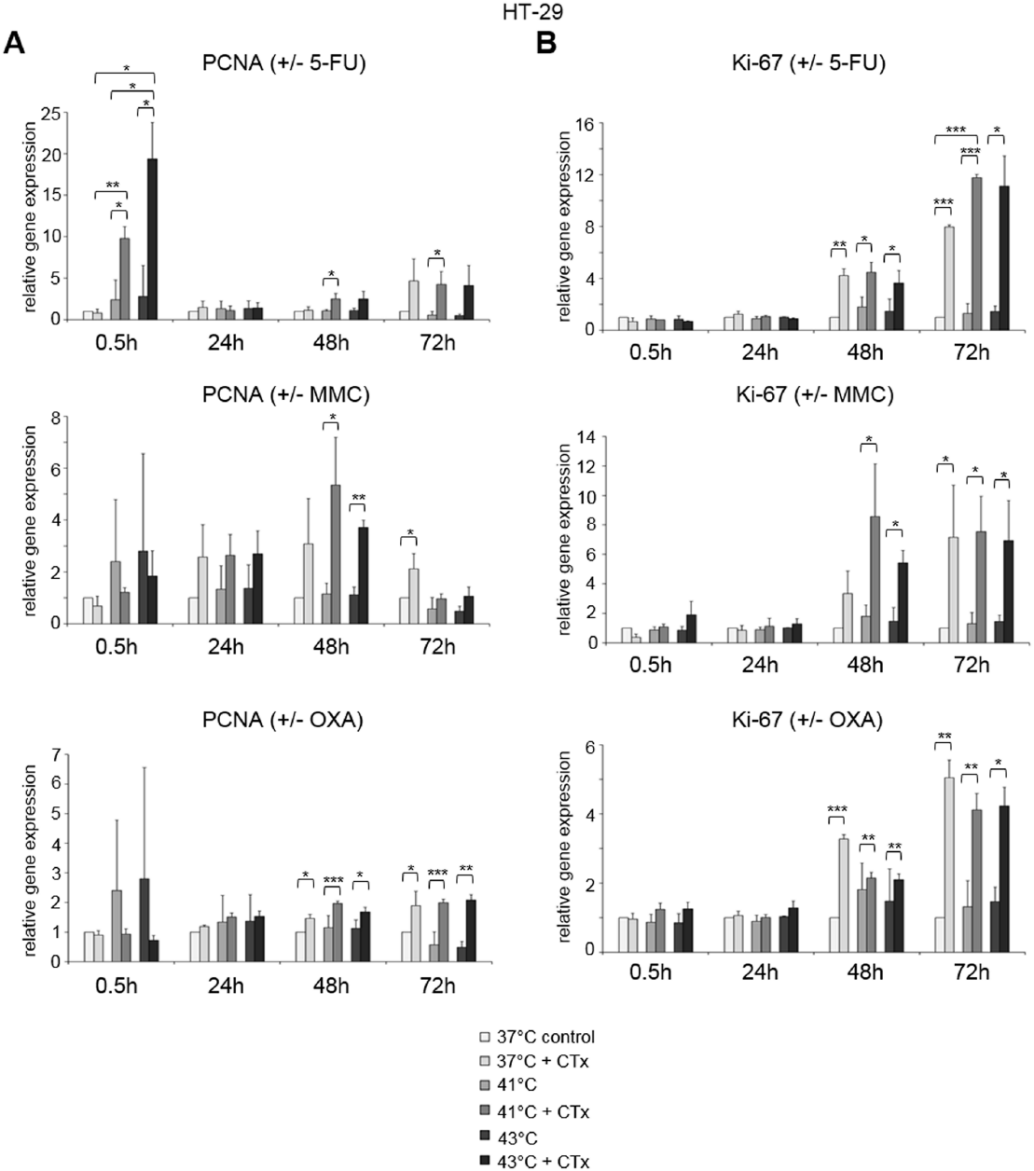
Gene expression analysis (real-time polymerase chain reaction) of proliferation markers (A) PCNA (proliferating cell nuclear antigen and (B) Ki-67 in HT-29 cells 0.5, 24, 48, and 72 hours after treatment (60 minutes) with hyperthermia including or without additional chemotherapy (CTx) using 5-fluorouracil (5-FU), mitomycin C (MMC), or oxaliplatin (OXA). Colon cancer cells under normothermic condition without cytostatic treatment (37°C control) were standardized to baseline. The relative gene expression is expressed as 2^−ΔΔCq^ (= fold difference, FD). Reproducibility was confirmed by 3 independent runs. *P < .05; **P < .0005; ***P < .0001 (Student *t* test).

Rapidly upregulated PCNA gene expression after hyperthermic chemotherapy with 5-FU was observed in HT-29 cancer cells, whereas no changes were visible after normothermic incubation with the substance (0.5 hours after treatment at 41°C: FD 9.8 and 43°C: FD 19.4) (Figure 7A, top). After 48 and 72 hours, cancer cells showed again elevated PCNA gene expression (41°C: FD 2.5 and FD 4.3, 43°C: FD 2.5 and FD 4.1, respectively). Upregulated PCNA gene expression was also detected in tumor cells incubated with 5-FU chemotherapy under normothermic condition (72 hours at 37°C: FD 4.6) (Figure 7A, top). Hyperthermic chemotherapy using MMC resulted in increased PCNA expression 24 and 48 hours after incubation (41°C: FD 2.6 and FD 5.3, 43°C: FD 2.7 and FD 3.7, respectively), whereas normothermic chemotherapy with MMC caused elevated PCNA expression after 24, 48, and 72 hours (FD 2.6, FD 3.1, and FD 2.1, respectively) (Figure 7A, center). In HT-29 cells treated with OXA, moderately increased PCNA gene expression was observed after 48 hours (41°C: FD 1.9 and 43°C: FD 1.7) and after 72 hours (37°C: FD 1.9, 41°C: FD 2.0, and 43°C: FD 3.0) (Figure 7A, bottom).

Next to PCNA, Ki-67 expression was analyzed as second proliferation marker. After 24 hours and more vigorously after 48 hours of initial normothermic and hyperthermic incubation with all 3 cytotoxic substances, HT-29 tumor cells demonstrated significantly upregulated Ki-67 expression (5-FU 37°C: FD 4.2 and 8.0, 41°C: FD 4.5 and 11.8, and 43°C: FD 3.6 and 11.1, respectively; MMC 37°C: FD 3.4 and 7.2, 41°C: FD 8.6 and 7.5, and 43°C: FD 5.4 and 6.9, respectively; OXA 37°C: FD 3.3 and 5.1, 41°C: FD 2.2 and FD 4.1, and 43°C: FD 2.1 and 4.2, respectively (Figure 7B)). Comparable results for PCNA (exception: OXA) and Ki-67 gene expression were obtained in SW480 (Supplementary Figure S7) and SW620 (Supplementary Figure S8) colon cancer cells.

**Figure 8.**
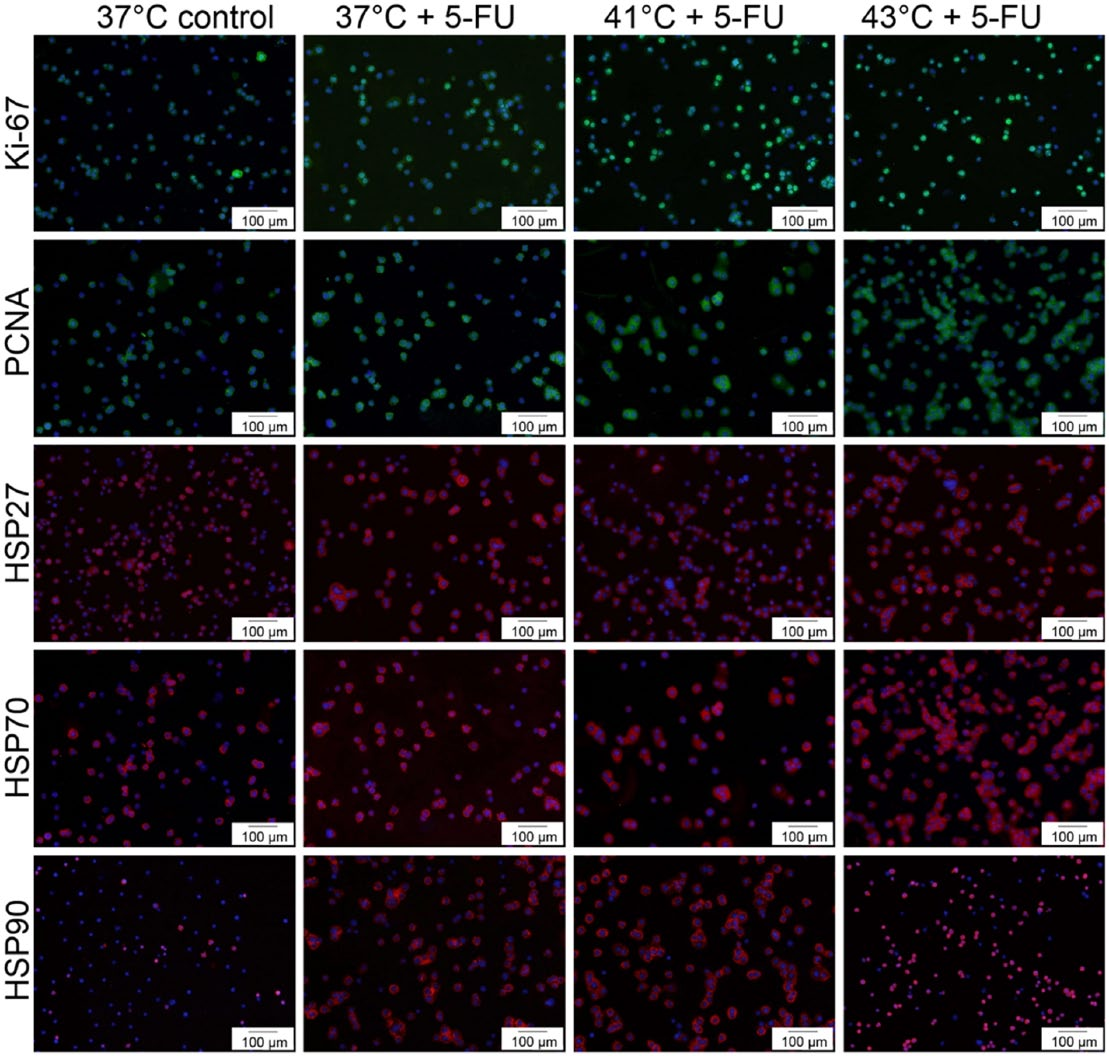
Immunofluorescent stainings of PCNA (green), Ki-67 (green), HSP27 (red), HSP70 (red), and HSP90 (red). FITC (fluorescein isothiocyanate), green; Cy3 (indocarbocyanine), red; DAPI (4′,6-diamidino-2-phenylindole dihydrochloride); blue—nuclear counterstaining.

To additionally visualize PCNA and Ki-67 expression increase after exposure to chemotherapy, representative examples of immunofluorescent stainings in SW480 cells treated with 5-FU at normothermia (37°C) and hyperthermia (41°C and 43°C) are demonstrated in Figure 8 (green fluorescence).

**Figure 9.**
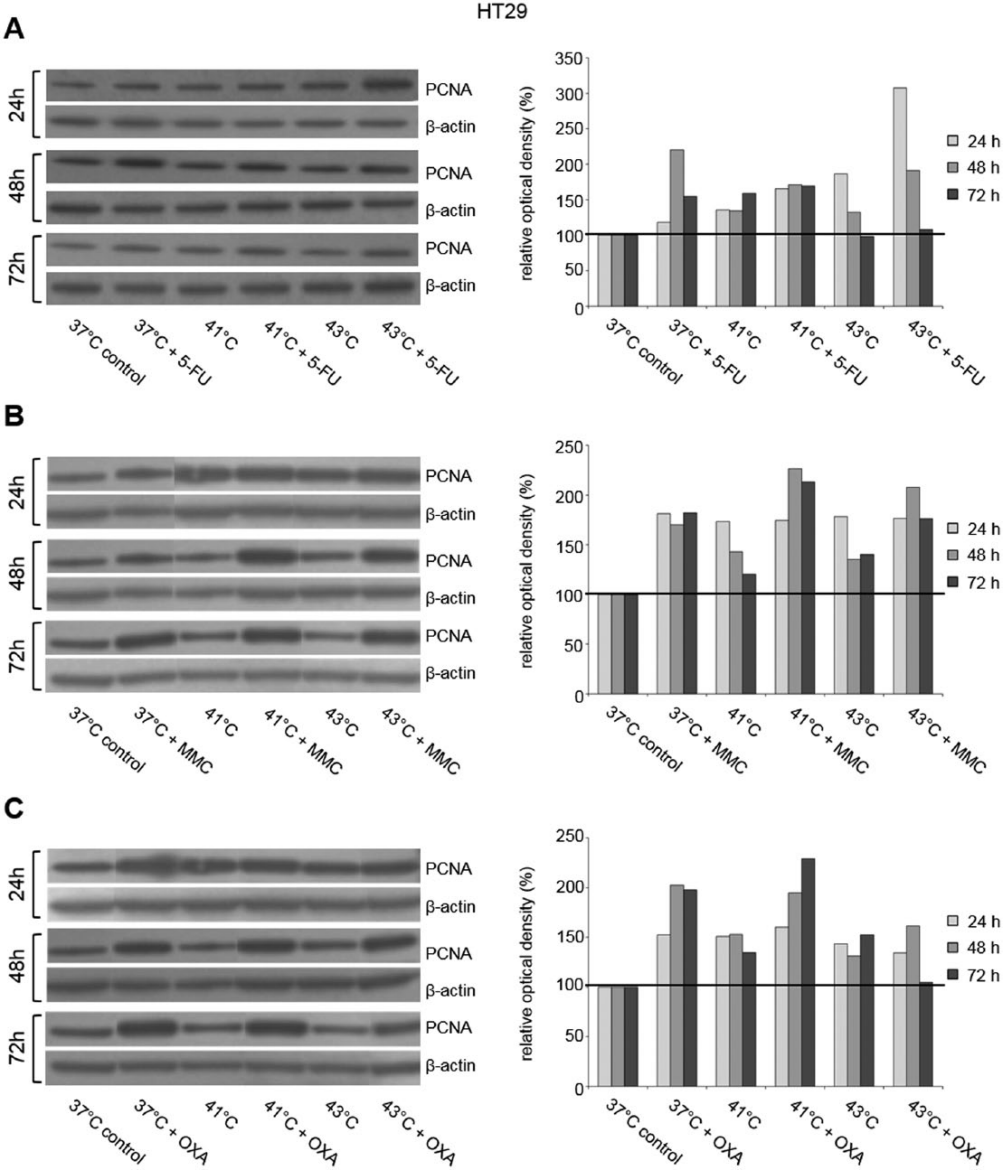
Western blot analysis of PCNA (proliferating cell nuclear antigen) in HT-29 cells 24, 48, and 72 hours after treatment (60 minutes) with (A) hyperthermia including or without additional chemotherapy using (B) 5-fluorouracil (5-FU), mitomycin C (MMC), or (C) oxaliplatin (OXA). β-actin probe was used as a control for protein loading. Relative optical density was determined using ImageJ software: values for proteins of interest were calculated in relation to values of loading controls. Normothermic cells without cytostatic treatment (37°C control) were standardized to baseline.

For PCNA, additional Western blot analysis was performed. In HT-29 cancer cells, 5-FU chemotherapy under normothermic condition resulted in moderately increased protein expression at all investigated time points (Figure 9A). Hyperthermic 5-FU chemotherapy resulted in increased protein expression after exposure to 41°C and 43°C (ROD = 307% and ROD = 191%, respectively) compared with normothermic controls and corresponding cancer cells without 5-FU chemotherapy (Figure 9A). Normothermic and hyperthermic treatment with MMC comparably induced PCNA expression at all investigated time points (Figure 9B). HT-29 tumor cells treated with OXA again demonstrated increased PCNA expression (Figure 9C). In accordance with these observations, comparable results were obtained in SW480 and SW620 colon cancer (Supplementary Figure S9).

### Antiapoptotic protein analysis after hyperthermic chemotherapy

**Figure 10.**
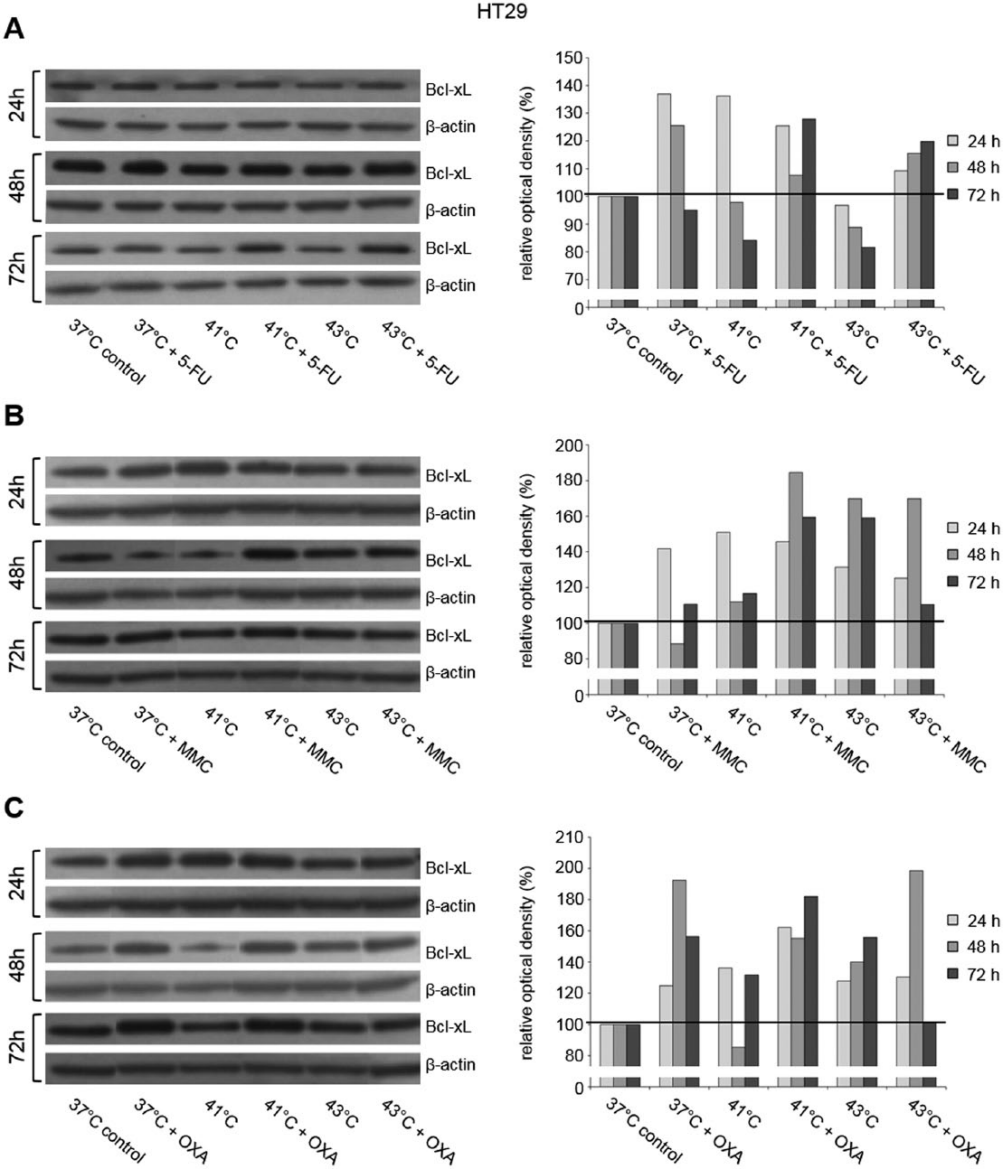
Western blot analysis of antiapoptotic Bcl-xL in HT-29 cells 24, 48, and 72 hours after treatment (60 minutes) with hyperthermia including or without additional chemotherapy using (A) 5-fluorouracil (5-FU), (B) mitomycin C (MMC), or (C) oxaliplatin (OXA). β-actin probe was used as a control for protein loading. Relative optical density was determined using ImageJ software: values for proteins of interest were calculated in relation to values of loading controls. Normothermic cells without cytostatic treatment (37°C control) were standardized to baseline.

To investigate the influence of hyperthermic chemotherapy on tumor cell apoptosis, protein expression of antiapoptotic Bcl-xL was further analyzed. As demonstrated in HT-29 cancer cells, initial treatment with 5-FU under normothermic and hyperthermic condition resulted in moderate Bcl-xL increase at all investigated time points (Figure 10A). Mitomycin C as well as OXA treatment also caused an induction of Bcl-xL expression after 24, 48, and 72 hours (Figure 7B and 7C).

### Bcl-xL and PCNA analysis after hyperthermic chemotherapy in patient tumors from PC of colorectal cancer origin

**Figure 11.**
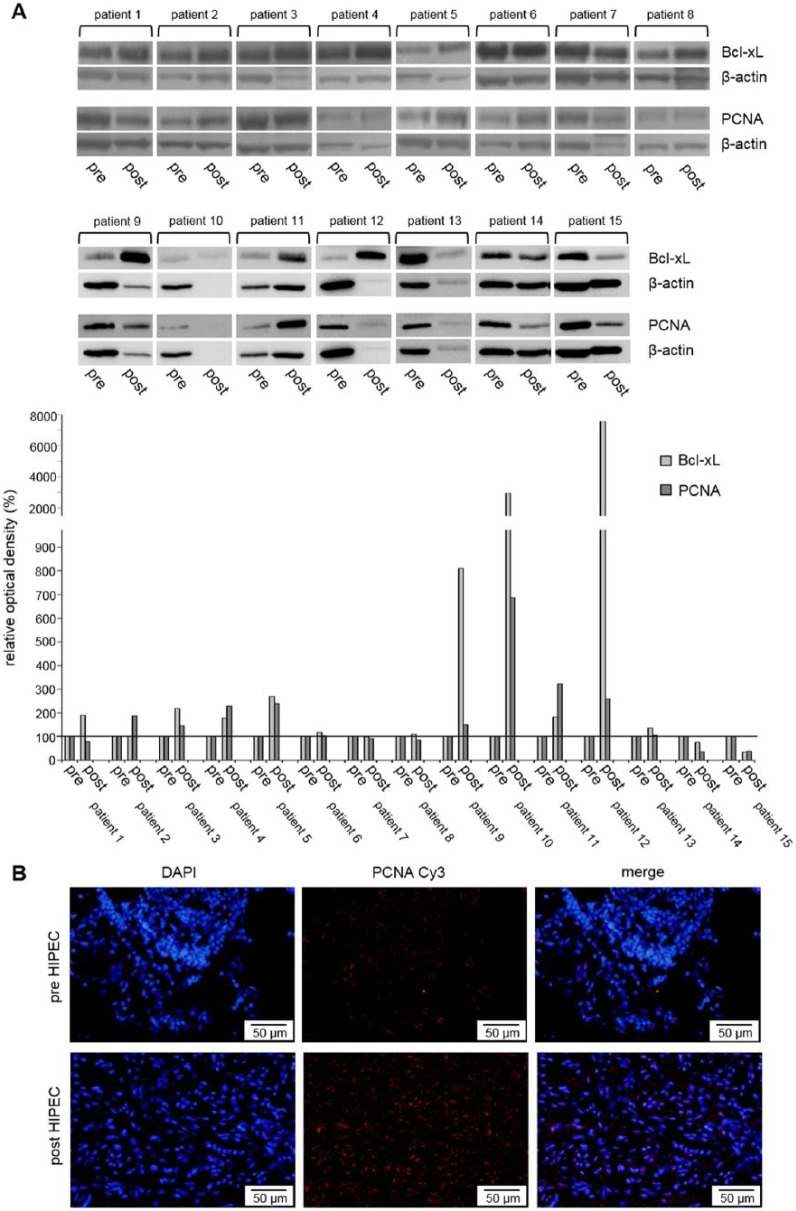
(A) Western blot analysis of antiapoptotic Bcl-xL and proliferation marker PCNA (proliferating cell nuclear antigen) in tumor tissue samples from peritoneal carcinomatosis of patients with colorectal cancer before (pre) and after (post) HIPEC. (B) Immunofluorescent staining of PCNA in peritoneal tumor tissue samples before (pre) and after (post) HIPEC. Cy3 (indocarbocyanine), red; DAPI (4′,6-diamidino-2-phenylindole dihydrochloride), blue—nuclear counterstaining. HIPEC indicates hyperthermic intraperitoneal chemotherapy. β-actin probe was used as a control for protein loading. Relative optical density was determined using ImageJ software: values for proteins of interest were calculated in relation to values of loading controls. Pre-HIPEC samples were standardized to baseline.

For Bcl-xL and PCNA, additional Western blot analysis was performed in tumors from 22 patients with PC undergoing a HIPEC procedure with MMC or OXA combined or without systemic 5-FU. After Western blot analysis, 7 of 22 patient tumor specimens had to be excluded from further analysis due to pronounced protein degradation in samples after HIPEC. Peritoneal tumors showed an increased expression for both Bcl-xL (n = 6/8, 75%, ROD = 190%, ROD = 218%, ROD = 228%, ROD = 269%, ROD = 119%, and ROD = 110%) and PCNA (n = 4/8, 50%, ROD = 187%, ROD = 146%, ROD = 228%, and ROD = 239%) (Figure 11A).

In addition, increased PCNA expression after a HIPEC procedure was demonstrated in the tumors using immunofluorescent techniques for the peritoneal tumors (Figure 11B).

## Discussion

Today, the combined treatment of CRS with HIPEC represents a promising therapeutic strategy in several peritoneal malignancies.^4^ Recent clinical data point to beneficial effects in selected patients with isolated PC and indication for this procedure. For patients with PC from colorectal cancer, HIPEC was recently reported to be associated with improved overall survival and disease-free survival.^6,26,27^ These reports were mostly from matched case control studies from single centers and protocols comprised the chemotherapeutic substances MMC and OXA that were given at high dosages locally into the abdomen for either 30 or 60 minutes. Hyperthermic conditions in the abdomen ranged between 41°C and 43°C. However, these protocols were mostly defined on empirical data. Thus, further (pre)clinical data may improve hyperthermic effects when combined with the currently used chemotherapeutic substances in the clinic. Combined hyperthermia with high-dose local chemotherapy has most recently been demonstrated to exert beneficial effects in several settings.^28^ One of the obvious mechanisms described to be responsible is an increase in the drug concentration in the peritoneal tissue as has been demonstrated in a murine model analyzing the effects of heat on 3 different doses of intraperitoneal OXA (460, 920, and 1840 mg/mL) at 3 different perfusion temperatures (37°C, 40°C, and 43°C).^29^ In addition, varying effects of hyperthermia considering perfusion and oxygenation have been detected in cancerous and normal tissue. Although perfusion is increased by hyperthermia in normal tissue, opposite observations were made in tumor tissues with reduced perfusion leading to hypoxia in the tumor cells.^30^ Moreover, functional proteins within the tumor cells become unfolded, and the cell membrane, nuclear DNA, cytoskeleton, and cell organelles can be damaged.^31^ Heat shock proteins are likely to play a significant role in this process as highly conserved proteins induced in response to different environmental, physical, and chemical stress factors such as cytotoxic agents and hyperthermic conditions. They act as a defense mechanism toward apoptosis-inducing events. Heat shock proteins are known to play a crucial role in chaperoning misfolded proteins and protecting cells from damage and overexpression of HSPs in various tumor entities in association with poor prognosis.^14,15,19,20^ Previously, we demonstrated upregulated HSP27, HSP70, and HSP90 mRNA and protein expression in tumors from PC of different entities after clinical HIPEC procedures. These observations were confirmed in in vitro studies analyzing colon cancer cells exposed to hyperthermia early after treatment (30 minutes and 12 hours).^21^

Here, we demonstrated for the first time that colon cancer cells exposed to cytostatic treatment with chemotherapeutic substances for HIPEC (MMC and OXA) and for systemic chemotherapy (5-FU) are highly sensitive for upregulated expression of all 3 HSPs, HSP27, HSP70 and HSP90, when imitating clinically HIPEC conditions. Cytostatic treatment for 1 hour using 5-FU, MMC, or OXA under hyperthermia resulted in significantly increased gene expression of investigated HSPs (HSP27, HSP70, and HSP90) compared with normothermic conditions (37°C) as well as corresponding hyperthermic conditions alone without chemotherapy. Even 3 days after exposure, increased HSP expression profiles were observed suggesting similar effects in vivo after a HIPEC procedure. This may occur particularly in those residual tumor cells from peritoneal surface areas of a patient with local hypoxia. Moreover, hyperthermic chemotherapy resulted in distinct upregulation of HSP expression combined with constitutively overexpressed HSP70 and HSP90 in the investigated colon cancer cells. Although HSP27 expression was detected at moderate basal levels, intense HSP70 and HSP90 baseline expression was observed in normothermic controls.

The analyzed HSP27, HSP70, and HSP90 expression profiles indicate prolonged intracellular repair mechanisms in a minor population of the colon cancer cells to survive the cellular stress exerted by hyperthermia and particularly cytostatic treatment. However, it should be taken into account that the investigated tumor cells have been kept under optimized conditions including fresh RPMI medium with fetal calf serum and constantly CO_2_ atmosphere following the hyperthermic chemotherapy. Under clinical circumstances, hypoxia and diminished blood supply in the abdomen may aggravate the HIPEC-induced intracellular stress with even more prolonged and vigorous HSP-mediated repair mechanisms in the tumor cells. Thus, remaining single or aggregated tumor cells that are detached from peritoneal surface metastases and are incompletely affected from a HIPEC procedure may survive through HSPs that circumvent tumor cell apoptosis under such circumstances.

To protect cells from such damage and apoptosis, HSPs interact with several survival and antiapoptotic pathways allowing the cell to deal with potentially lethal conditions.^16^ HSP27 has been shown to interact and inhibit components of both extrinsic and intrinsic apoptotic pathways.^32^ The extrinsic apoptotic pathway is blocked by the inhibition of tBid (truncated BID), a membrane-targeted death ligand that induces release of cytochrome c from the mitochondria. Moreover, binding of HSP27 to DAXX (death domain–associated protein) prevents transmission of the extrinsic signal to proapoptotic Bcl-2.^33^ In addition, HSP27 binds to cytochrome c itself thus suppressing the progress of apoptosis through inhibition of the interaction with Apaf-1 (apoptotic peptidase–activating factor 1) and the following activation of caspase 9 and assembly of the apoptosome.^34,35^ It also disables the release of Smac/Diablo and their inhibition of IAPs (inhibitors of apoptosis).^36^ Moreover, HSP27 inhibits apoptosis by regulating upstream signaling pathways. Survival factors, such as the platelet-derived growth factor, inhibit apoptosis by activating the PI3K/Akt (phosphatidylinositol 3-kinase/protein kinase B) pathway. Akt targets multiple proteins of apoptotic signaling pathways, including Bad (Bcl-2–associated agonist of cell death) and caspase-9, and HSP27 has been shown to bind Akt and cause its activation in stressed cells.^32,37^

HSP70 also enhances the ability of cells to suppress apoptosis under potentially lethal conditions. The extrinsic pathway of apoptosis is blocked twice by the interaction of HSP70 with ASK (apoptosis signal-regulating kinase) and JNK (c-Jun N-terminal kinase).^38^ It inhibits the stress-induced dephosphorylation of JNK and abides the level of inactive JNK. JNK is involved in the regulation of various proteins within apoptotic signaling pathways, eg, the protooncogene c-Myc, the tumor suppressor p53, and Bcl2, all involved in permeabilization of the mitochondrial membrane.^39,40^ Moreover, the cleavage of Bid to tBid and the following release of cytochrome c factors into the cytoplasm are inhibited by HSP70.^41^ In addition, by blockade of Apaf-1, HSP70 inhibits formation of the apoptosome.^42^

Similar to HSP27 and HSP70, HSP90 also exerts antiapoptotic functions to support cellular survival mechanisms. Just as HSP70, HSP90 directly interacts with Apaf-1 and inhibits the formation of a functional apoptosome and the recruitment and activation of procaspase 9.^43^ HSP90 also interferes with the death receptor/nuclear factor κB (NF-κB) signaling cascade by binding to RIP (receptor-interacting protein) which interacts with the death receptors of the TNFR (tumor necrosis factor receptor) family. This interaction provokes an increased activation of IKK (IκB kinase complex) and thus inactivation of IκB (inhibitor of NF-κB). Consequently, NF-κB can serve as an active transcription factor for several antiapoptotic target proteins such as Bcl-xL.^44^

Interestingly, hyperthermic chemotherapy resembling HIPEC-like conditions in the clinic induced expression of antiapoptotic Bcl-xL as shown in the investigated colon cancer cells and confirmed in peritoneal metastatic tumor tissues from patients with colorectal cancer undergoing HIPEC procedures. Moreover, an increased “chemoresistance” was observed in colon cancer cells treated with hyperthermic chemotherapy compared with normothermic incubation with 5-FU, MMC, and OXA. Remarkably, proliferation markers, PCNA and Ki-67, were induced in these tumor cells exposed to chemotherapy which was in case of PCNA confirmed in peritoneal tumor tissues after clinical HIPEC procedures. The results indicate that a minor population of incompletely damaged and robust tumor cells was responsible for the observed increase in expression of proliferation markers and that this phenomenon was attributed to a rebound effect during first days after hyperthermic chemotherapy. In addition, results from MTS assays were in line with those from tumor cell proliferation markers and demonstrated increased tumor cell viability in samples exposed to hyperthermic chemotherapy compared with normothermic treatment. Consequently, these tumor cells survived the hyperthermic and chemotoxic stress.

Based on their interference with apoptotic pathways, HSPs have become an interesting target for inhibitory strategies in cancer therapy. Many potential inhibitors of particularly HSP90 have been tested for cancer therapy, and currently, HSP90 inhibitors are being evaluated in numerous clinical trials.^45^ Next to STA-9090, AUY922 is in development, and the combination of AUY922 with 5-FU and OXA has been described to show a synergistic antiproliferative effect in gastric cancer.^45,46^ As HSP90 inhibition has been demonstrated to induce the expression of HSP70, dual targeting of HSP70 and HSP90 might be beneficial to successfully inhibit HSP-dependent antiapoptotic mechanisms.^47^ Different small-molecule HSP70 inhibitors such as VER155008 and 2-phenylethynesulfonamide have already been proven to enhance the cytotoxicity of HSP90 inhibitors.^47–50^ For targeting HSP27, 2 small-molecule inhibitors, quercetin and RP101, are under development. Similar to AUY922, quercetin potentiates the effects of many first-line chemotherapeutics including doxorubicin, cisplatin, gemcitabine, and 5-FU.^51–53^ Interestingly, the herein presented cell viability data from HSP inhibition assays, using a combination of HSP90 (17-AAG) and HSP70 (VER155008) inhibitors, indeed demonstrated significantly reduced viability in our colon cancer cells previously exposed to hyperthermic chemotherapy, suggesting a beneficial effect of HSP inhibition after HIPEC therapy.

In summary, our findings from in vitro mechanistic studies as well as investigation of peritoneal colon cancer metastases after a HIPEC procedure indicate that induction of HSP27, HSP70, and HSP90 expression through hyperthermic and cytostatic stress in colon cancer cells actively inhibits apoptosis-inducing effects during and after the exposure to HIPEC-induced stress. In addition, cancer cells that were able to survive the hyperthermic and chemotoxic damage demonstrated increased cell viability and proliferation.

## Supplementary Material

Supplementary material
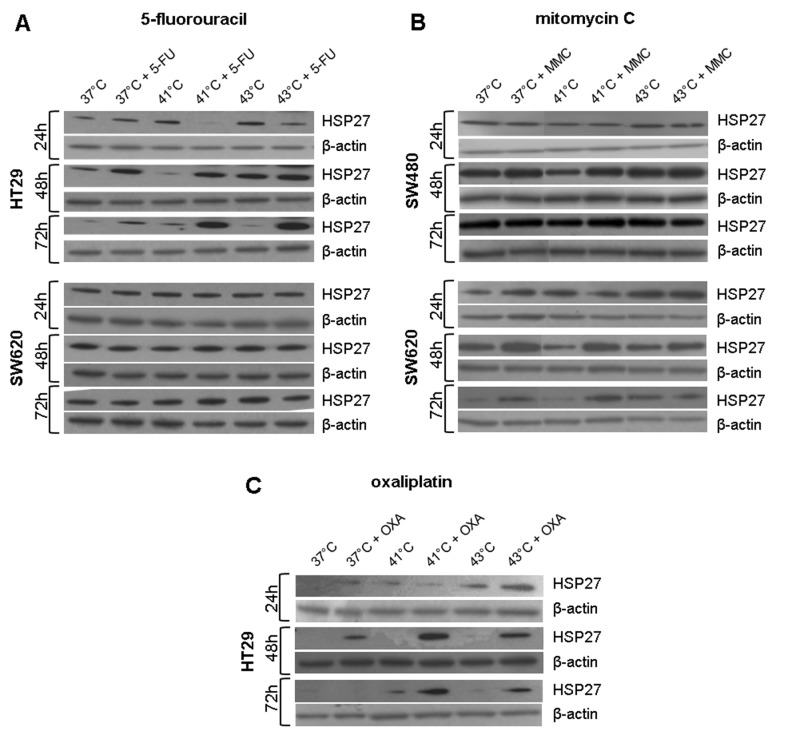

Supplementary material
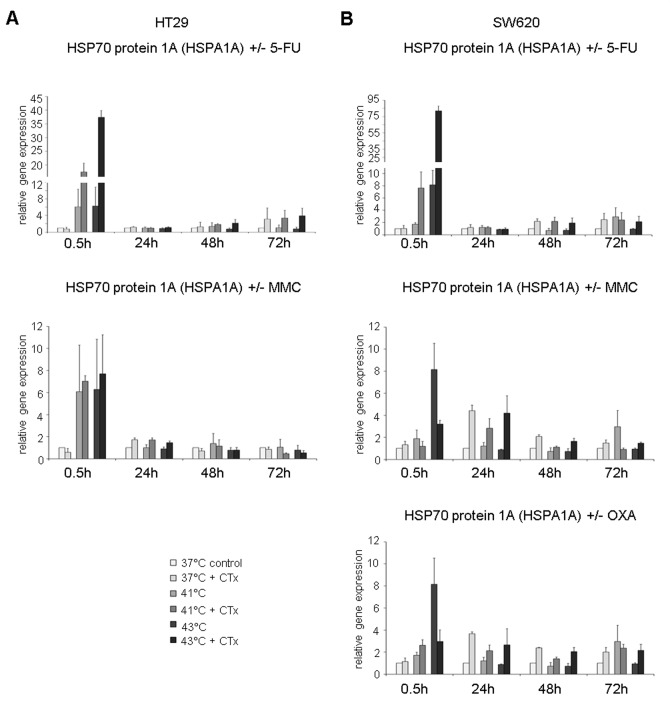

Supplementary material
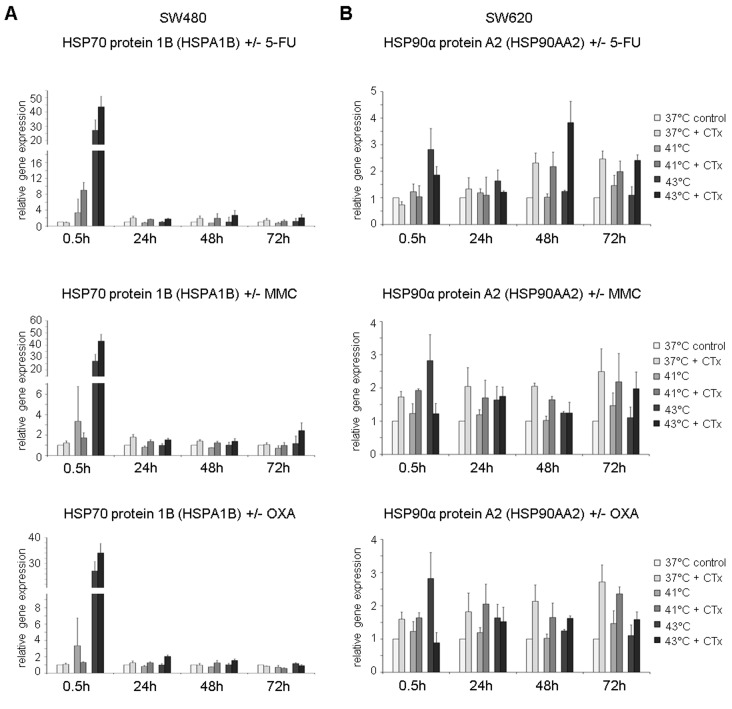

Supplementary material
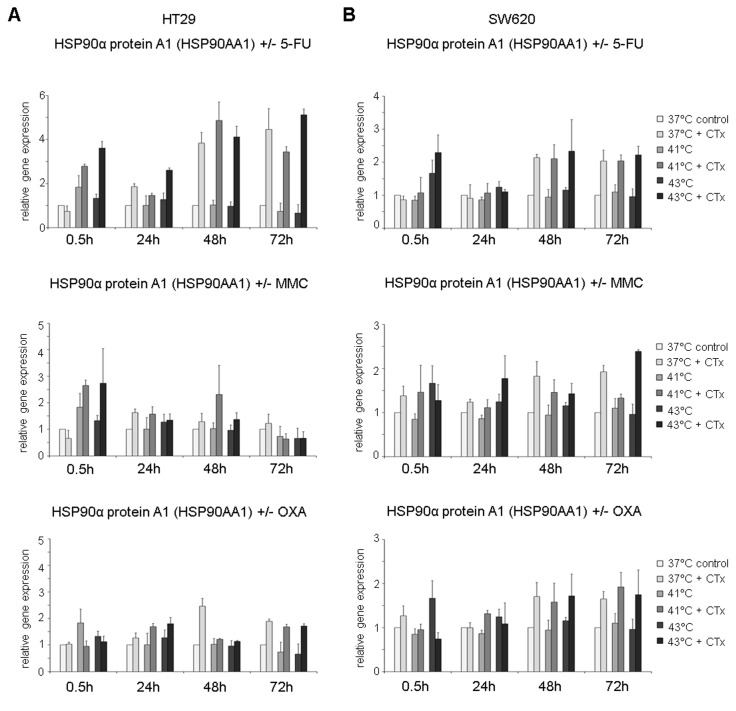

Supplementary material
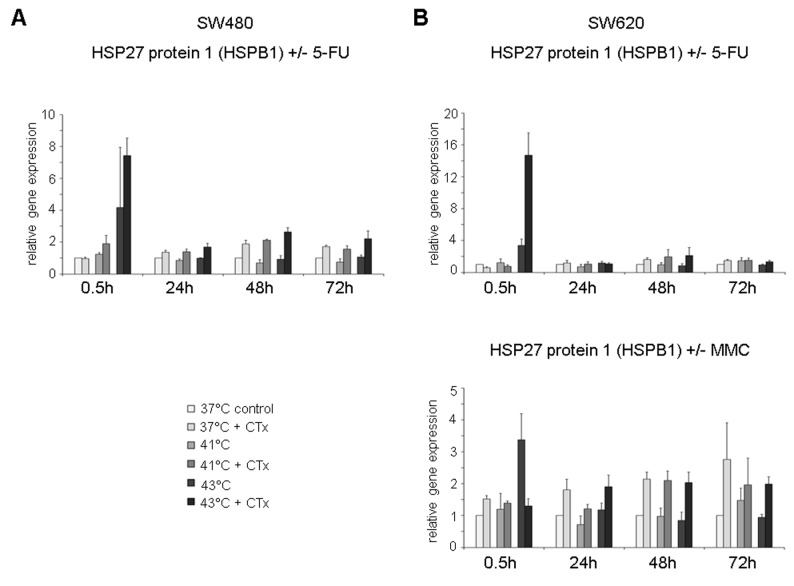

Supplementary material
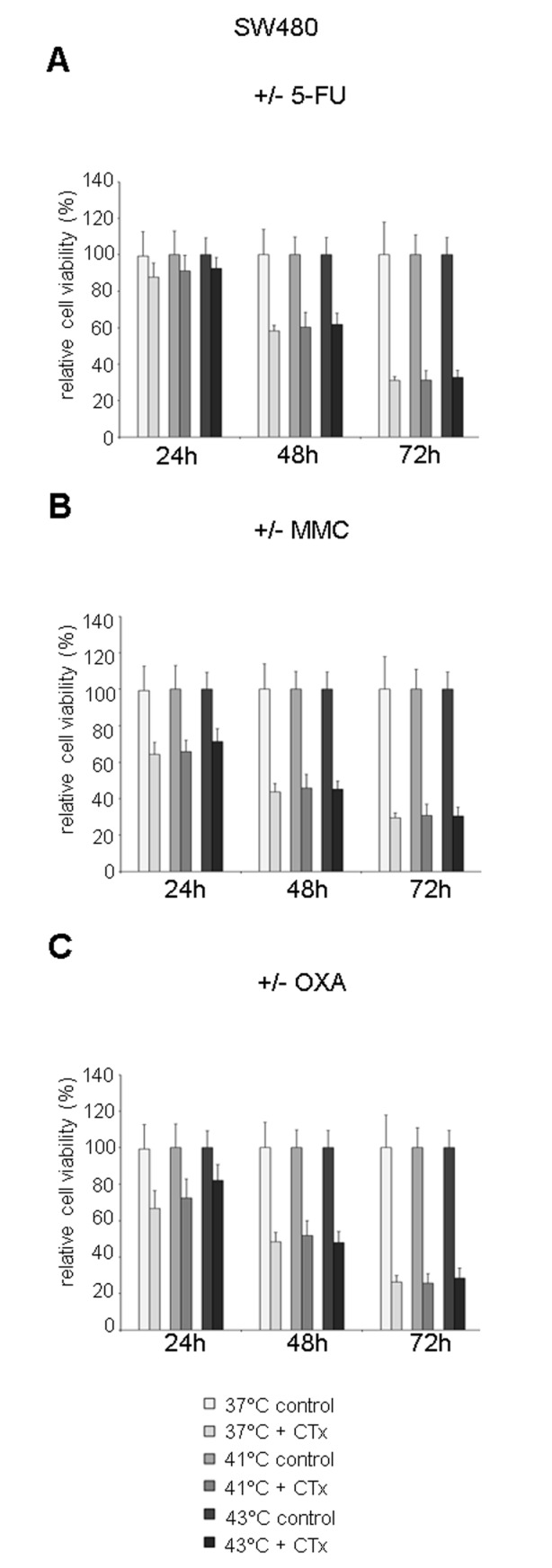

Supplementary material
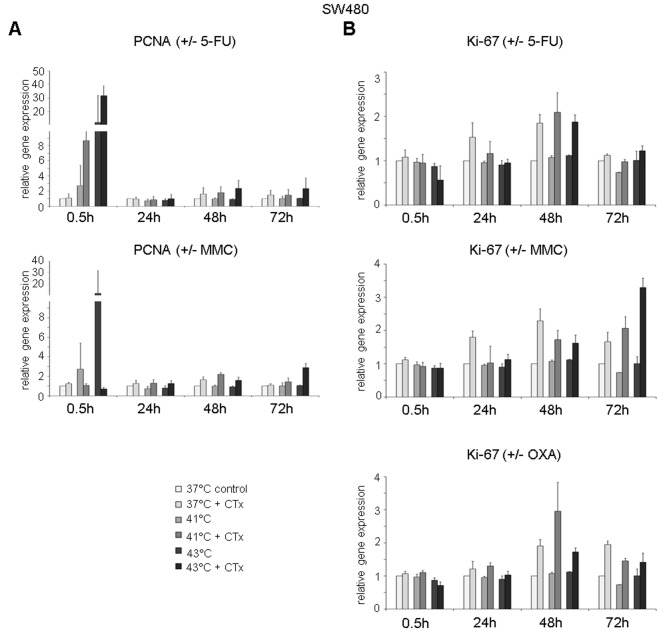

Supplementary material
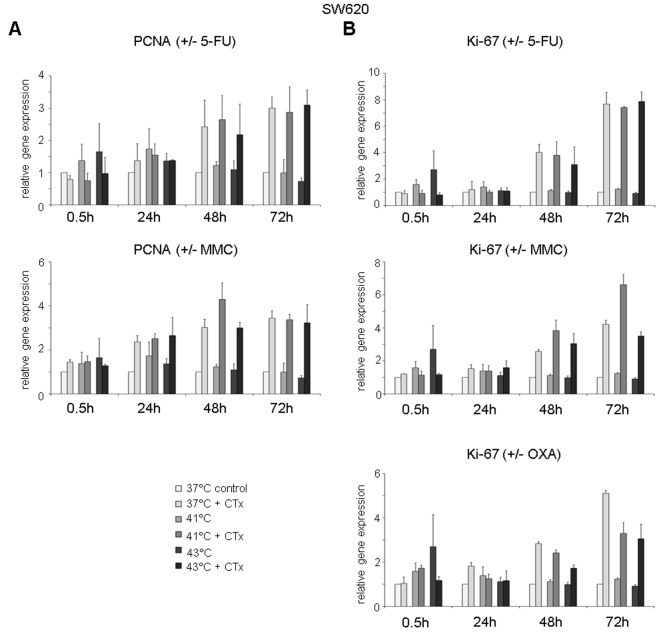

Supplementary material